# Pediatric Applications of Digital Therapeutics: Clinical Evidence and Implementation Landscape

**DOI:** 10.7759/cureus.91592

**Published:** 2025-09-04

**Authors:** Venkata Sushma Chamarthi

**Affiliations:** 1 Department of Pediatrics, Valley Children’s Healthcare, Fresno, USA

**Keywords:** amblyopia, attention-deficit/hyperactivity disorder (adhd), digital therapeutics, pediatrics, virtual reality

## Abstract

Digital therapeutics (DTx) are evidence-based software interventions designed to prevent, manage, or treat medical conditions through validated therapeutic mechanisms of action. They are emerging as a transformative paradigm in pediatric healthcare, where accessibility, engagement, and personalization are particularly critical. This review examines the landscape of prescription digital therapeutics for children and adolescents, focusing on current applications, regulatory considerations, implementation challenges, and future innovations. Although only a limited number of prescription digital therapeutics have been formally authorized for pediatric use, early examples demonstrate feasibility and therapeutic benefit for conditions such as attention-deficit/hyperactivity disorder (ADHD) and amblyopia. Beyond these, research is rapidly expanding into a broader set of conditions, including autism spectrum disorders, anxiety, substance use prevention, and management of chronic illnesses, highlighting the potential of digital therapeutics to complement traditional treatment approaches in specific contexts. Despite this promise, several barriers to implementation remain, with access constrained by the digital divide, socioeconomic disparities, and variability in insurance reimbursement. Additional challenges include ensuring developmental appropriateness, addressing privacy and safety concerns specific to children, and creating sustainable clinical integration pathways. Looking ahead, emerging technologies such as artificial intelligence-driven personalization, immersive virtual and augmented reality environments, and integration with wearable devices offer opportunities to overcome current limitations. Pediatric digital therapeutics hold promise as tools that may advance precision medicine, though current evidence is limited mainly to a few conditions, such as ADHD and amblyopia. With the potential to deliver scalable, cost-effective, and engaging interventions that expand access to high-quality care for children worldwide.

## Introduction and background

Digital therapeutics (DTx) represent a paradigm shift in pediatric healthcare delivery, offering evidence-based software interventions that provide therapeutic mechanisms of action to prevent, manage, or treat medical disorders [1,2]. Unlike general wellness applications, prescription digital therapeutics undergo formal clinical validation and regulatory review, though the approval requirements and timelines are typically less extensive than those for traditional pharmaceuticals [2]. For pediatric populations, DTx addresses critical gaps in healthcare access while offering significant advantages, including real-time adaptation to individual patient responses, continuous monitoring capabilities, and the potential to scale evidence-based interventions across underserved populations [3].

The pediatric DTx landscape faces unique challenges, including developmental appropriateness requirements, parental involvement considerations, and specialized safety monitoring needs for vulnerable populations. Current market projections indicate rapid growth, with Lindus Health (2024) estimating the overall DTx sector will expand from $4-7 billion in 2023 to $10.1 billion in 2024-a faster trajectory than most independent forecasts, which typically place this milestone later in the decade [4]. Pediatric applications represent a growing segment driven by increasing recognition of unmet healthcare needs, technological advances enabling age-appropriate interventions, and expanding regulatory pathways supporting innovation. Developmental differences further shape DTx design and implementation. Younger children often require caregiver involvement and simplified interfaces, while school-aged children respond well to gamified, interactive features. Adolescents and young adults benefit from greater autonomy, but also raise considerations of privacy, adherence, and stigma.

In this review, we adopt a clinical-regulatory lens to analyze pediatric digital therapeutics. Our unique perspective lies in synthesizing efficacy data from clinical trials alongside regulatory milestones, implementation barriers, and policy considerations. This integrated framework highlights how pediatric DTx differ from adult-focused digital health tools, particularly in terms of developmental appropriateness, parental involvement, and specialized safety requirements. By framing the evidence within this dual clinical and regulatory context, the review aims to inform both clinicians and policymakers about the current state of pediatric DTx, their limitations, and its potential trajectory within precision medicine.

Methods

This review was conducted as a targeted narrative synthesis of published literature on pediatric digital DTx. PubMed and regulatory agency sources, including the U.S. Food and Drug Administration (FDA) and the European Medicines Agency (EMA), were searched for studies published between 2010 and 2025 using combinations of keywords such as “digital therapeutics”, “pediatrics”, “children”, “adolescents”, “ADHD”, “autism”, “anxiety”, and “chronic disease”. In addition to scientific articles, FDA approval summaries and device databases (De Novo and 510(k)) were examined for pediatric-specific regulatory decisions, while EMA documents were reviewed to provide an international comparison. Priority was given to peer-reviewed randomized controlled trials, systematic reviews, meta-analyses, and official regulatory documents. Industry reports and white papers were considered only to provide market and pipeline context. Studies were included if they reported clinical outcomes, safety data, or regulatory milestones relevant to pediatric populations (ages 0-18). Non-prescription wellness applications and adult-only studies were excluded. Evidence was organized by therapeutic domain (neurodevelopmental, mental health, and chronic disease) to highlight patterns of efficacy, safety, and regulatory precedent. Given the targeted nature of this review, studies most pertinent to the clinical and regulatory focus of the article were selected and synthesized.

## Review

Current applications by therapeutic area

Attention-Deficit Hyperactivity Disorder

EndeavorRx (Akili Interactive) represents the most clinically validated pediatric DTx, serving as the first FDA-approved DTx for any pediatric indication. Initially approved for children ages 8-12 years in June 2020 via the FDA De Novo pathway, EndeavorRx received label expansion to ages 8-17 in December 2023 based on clinical studies involving 162 adolescents. The therapeutic mechanism operates through proprietary algorithms that continuously adapt difficulty levels based on individual performance metrics, ensuring optimal cognitive challenge throughout treatment. EndeavorRx delivers therapeutic intervention through video game-based technology utilizing the Selective Stimulus Management Engine (SSME™). The intervention targets attention function in children with primarily inattentive or combined-type attention-deficit/hyperactivity disorder (ADHD) through 25-minute daily sessions requiring sustained attention and cognitive flexibility [1,2,5]. Treatment protocols typically involve four weeks of daily engagement, with some patients showing measurable improvements within the first two weeks of consistent use. Additional ADHD digital interventions include MindPro1, showing significant improvements in ADHD symptoms and Test of Variables of Attention (TOVA) attention scores in clinical trials with 52 children, demonstrating efficacy and a 100% parent acceptance rate [6]. The platform incorporates real-time biometric monitoring to track engagement levels and adjust therapeutic parameters accordingly. Various cognitive training platforms demonstrate moderate effect sizes for attention improvement (Cohen's d = 0.20 to 0.40) [7].

Autism Spectrum Disorder

DTx for autism focuses primarily on social skills training, communication enhancement, and behavioral intervention. Systematic reviews and meta-analyses of randomized controlled trials have reported small-to-moderate effect sizes for digital interventions targeting social-emotional skills in children with autism spectrum disorders [8]. Computer-based interventions have shown good outcomes, with results generally comparable to those of tablet- and smartphone-based applications [8,9]. The therapeutic approaches often incorporate gamification elements and personalized avatars to increase engagement and reduce anxiety associated with social learning. Key applications include Superpower Glass, utilizing artificial intelligence (AI)-driven wearable technology for facial engagement and emotion recognition [10], and various augmentative and alternative communication (AAC) platforms, including Proloquo2Go and TouchChat HD [11]. These platforms integrate machine learning algorithms that adapt to individual communication patterns and preferences over time. Social Story Creator applications and virtual reality-based social skills training platforms demonstrate promising preliminary results, particularly in controlled environment simulations that allow repeated practice of challenging social scenarios [11].

Anxiety and Mood Disorders

Digital cognitive behavioral therapy (CBT)-based interventions show significant efficacy for pediatric anxiety disorders. Meta-analyses indicate medium to large effect sizes for digital mental health interventions targeting social anxiety, with guided interventions showing superior outcomes to unguided approaches [12]. The structured nature of digital CBT allows for consistent delivery of evidence-based therapeutic techniques while maintaining treatment fidelity across diverse clinical settings. SparkRx (Limbix Health) received emergency use authorization during COVID-19 for adolescents and young adults ages 13-22 with depressive symptoms. While this authorization extended beyond the pediatric age range, the adolescent subgroup (ages 13-17) is relevant to pediatrics. The program combined CBT and behavioral activation protocols in a 5-week format, though long-term effectiveness data remain limited. The platform incorporates mood tracking features and automated check-ins to monitor patient progress and identify potential safety concerns. Additional applications include Mightier, utilizing video games with biofeedback for emotional regulation, and various mindfulness-based interventions adapted for pediatric populations [12]. These interventions often provide caregiver portals that summarize child progress while maintaining appropriate privacy safeguards.

Substance use Prevention and Treatment

Digital interventions for adolescent substance use focus primarily on prevention and early intervention. A rapid review of digital interventions identified primarily personalized feedback approaches targeting alcohol, tobacco, cannabis, and opioid use [13]. Effect sizes for substance use interventions remain small but significant, with meta-analyses showing modest reductions in weekly alcohol consumption. The interventions typically incorporate peer comparison data and real-time risk assessment tools to provide contextually relevant feedback to users. Prevention programs integrated with educational settings show particular promise for reaching at-risk populations, especially when combined with teacher training and family engagement strategies, whereas treatment-focused digital interventions for substance use disorders have generally demonstrated weaker outcomes. Many platforms utilize anonymous peer support networks and gamified goal-setting features to maintain long-term engagement in recovery-focused activities.

Chronic Disease Management

Asthma Management: Digital health interventions for other pediatric conditions, such as asthma management platforms combining smart inhaler sensors with mobile applications, show improved medication adherence and clinical outcomes according to systematic reviews, though evidence for reduced emergency department visits requires further validation in pediatric populations [14]. These platforms often include environmental trigger tracking and personalized action plan reminders that adapt to seasonal patterns and individual risk factors. Integration with wearable devices allows for continuous monitoring of activity levels and early detection of potential exacerbation triggers.

Diabetes Management:* *Technology integration in pediatric chronic disease management shows mixed but promising results across multiple conditions. In diabetes management, artificial pancreas systems and continuous glucose monitoring (CGM) have demonstrated clinically meaningful improvements in time-in-range, with studies showing 12% more time in the target glucose range (approximately three additional hours per day) and 26% improvement in nighttime control compared to standard care in children with Type 1 diabetes [15,16]. Real-world data from over 3,200 pediatric patients using advanced hybrid closed-loop systems achieved an average time-in-range of 74%, exceeding clinical consensus guidelines [17]. The systems incorporate predictive algorithms that anticipate glucose trends and automatically adjust insulin delivery to prevent both hyperglycemic and hypoglycemic episodes. However, mobile health applications show more variable results, as demonstrated by a 12-month randomized controlled trial of the Bant App in 92 adolescents ages 11-16 with Type 1 diabetes, which found no significant differences in primary clinical outcomes between intervention and control groups, though exploratory analysis suggested potential benefits for highly engaged users who performed frequent blood glucose monitoring, though these findings were not statistically significant and cannot be considered definitive evidence [18]. The variability in outcomes highlights the importance of user engagement strategies and the need for personalized intervention approaches in pediatric chronic disease management.

Beyond traditional chronic conditions, digital therapeutics may also support children recovering from prolonged critical illness, a population at risk for developing pediatric post-intensive care syndrome (PICS). PICS is characterized by persistent inflammation, immunosuppression, catabolism, and long-term sequelae, including physical, cognitive, and psychological morbidity [19]. Digital platforms could provide scalable tools for rehabilitation, long-term monitoring, and caregiver engagement in this high-risk group, complementing existing post-ICU follow-up models.

Clinical evidence and outcomes data

Efficacy Measurements and Statistical Significance

Clinical evidence supporting pediatric digital therapeutics spans multiple study designs, including randomized controlled trials, pilot studies, and real-world evidence. The diversity of study methodologies reflects the evolving nature of digital health research and the need to capture both controlled efficacy data and real-world effectiveness measures. Below is a summary of key clinical trials and outcomes across therapeutic areas (Table 1).

**Table 1:** Clinical Evidence Summary for Pediatric Digital Therapeutics Across Therapeutic Areas This table summarizes peer-reviewed clinical evidence supporting digital therapeutics (DTx) across pediatric populations with diverse developmental stages and clinical conditions. ADHD: attention-deficit hyperactivity disorder; RCT: randomized controlled trial; TOVA: test of variables of attention; FDA: Food and Drug Administration; DTx: digital therapeutics; SNAP-IV: Swanson, Nolan and Pelham-IV questionnaire; CBT: cognitive behavioral therapy; VR: virtual reality; AR: augmented reality; CGM: continuous glucose monitoring; AI: artificial intelligence

Intervention [Ref#]	Condition	Age Range	Study Design	Primary Outcome	Clinical Significance
EndeavorRx [1,2,5]	ADHD (primarily inattentive or combined type)	8–17 years	RCT; FDA De Novo authorization (2020) with label expansion (2023)	TOVA improvement	First FDA-approved DTx for pediatric use; significant improvement in attention
MindPro1 [6]	ADHD	6–12 years	Clinical trial	SNAP-IV improvement	Significant symptom reduction; comparable to FDA-approved DTx
Superpower Glass [10]	Autism Spectrum Disorder	Children	RCT	Social engagement	Significant improvement in socialization and gaze patterns
Digital CBT [12]	Anxiety disorders	7–18 years	Meta-analysis of RCTs	Symptom reduction	Medium–large effect sizes; guided > unguided
Substance Use Digital Interventions [13]	Substance use prevention/treatment	Adolescents	Rapid review	Substance use frequency reduction	Small but significant reductions in alcohol/tobacco/cannabis use
Smart Inhaler Platforms [14]	Asthma management	Children/adolescents	Systematic review	Medication adherence	Improved adherence; mixed evidence on ED visit reduction
Hybrid Closed-Loop Systems [15,16,17]	Type 1 Diabetes	Children/adolescents	RCT & real-world data	Time-in-range	+12% TIR; +26% nighttime control; 74% TIR in real-world cohort
Bant App [18]	Type 1 Diabetes	11–16 years	RCT	HbA1c change	No significant primary outcome change; potential benefit for engaged users
Luminopia One [20,21]	Amblyopia	4–12 years	RCT & registry data	Visual acuity improvement	First FDA-cleared treatment for 8–12 years in >20 years; 62% vs. 33% response rate
VR-Based DTx (32,33)	Procedural anxiety, social skills	Children/adolescents	Pilot trials & feasibility studies	Anxiety reduction, social engagement	Positive early results; potential for immersive skill training

Standardized Assessment Tools

Clinical trials utilize validated pediatric assessment instruments, including the TOVA® for ADHD, Vineland Adaptive Behavior Scales for autism, Childhood Anxiety Control Test for anxiety disorders, and medication adherence monitoring for chronic conditions. These objective measures enable precise efficacy determination and regulatory approval. The selection of appropriate outcome measures remains critical for demonstrating clinically meaningful improvements and ensuring regulatory acceptance. Many trials incorporate both primary clinical endpoints and secondary quality-of-life measures to capture comprehensive treatment effects.

Safety Profiles and Adverse Events

Safety data across pediatric DTx demonstrate favorable risk-benefit profiles. EndeavorRx trials reported zero serious adverse events, with treatment-related adverse events occurring in less than 5% of participants (primarily frustration and mild headache). Most pediatric DTx trials to date have reported adverse event rates below 10%, with no long-term safety concerns identified. However, these findings are primarily based on short-term studies, such as the EndeavorRx trial, and broader long-term safety data are still emerging [1]. The low adverse event rates likely reflect the non-invasive nature of digital interventions compared to pharmacological treatments. Pediatric-specific safety considerations include developmental appropriateness assessments, monitoring of screen time, and enhanced post-market surveillance requirements. The FDA requires additional safety data and extended monitoring for pediatric populations due to ongoing development and potential long-term effects. Long-term safety studies are particularly important given the potential for extended use periods and the developing nature of pediatric neurocognitive systems. Beyond adverse events, concerns also include the potential neurodevelopmental consequences of prolonged digital exposure, uncertainties regarding data privacy and protection in minors, and risks of overreliance on screen-based interventions. In addition, the increasing integration of AI-driven personalization in pediatric DTx raises ethical questions about transparency, algorithmic bias, and the vulnerability of children as a user group. Addressing these issues will require multi-stakeholder collaboration, including regulators, clinicians, ethicists, and patient families, to ensure that safety evaluations encompass not only short-term adverse events but also long-term developmental and ethical implications.

Regulatory landscape and FDA-approved pediatric digital therapeutics

Current FDA-Approved Pediatric DTx

EndeavorRx (Akili Interactive): Approved via De Novo pathway (DEN200026) in June 2020, representing the first FDA-authorized digital therapeutic for any pediatric condition. The approval created a new device classification for game-based digital therapeutics, requiring special controls, including clinical data demonstrating safety and effectiveness for attention improvement in ADHD [2]. The regulatory precedent established by EndeavorRx has paved the way for subsequent pediatric DTx approvals and provided a framework for clinical trial design and regulatory submission requirements. The device classification now serves as a predicate for future 510(k) submissions in similar therapeutic areas.

Luminopia One (Luminopia): Initially approved via the De Novo pathway in October 2021 for amblyopia treatment in children ages 4-7 years, with FDA clearance expanded to ages 4-12 in April 2025 based on real-world evidence from the PUPiL Registry. This expansion represents the first FDA clearance for an amblyopia treatment in the 8-12 age group in over 20 years, demonstrating 62% treatment response compared to 33% control group response in the VIEWS clinical trial [20,21]. The successful label expansion based on real-world evidence demonstrates the FDA’s evolving approach to post-market data collection and its willingness to consider registry data for regulatory decisions.

Regulatory Pathways and Requirements

Both pediatric DTx approvals utilized the De Novo pathway for novel devices without legally marketed predicates, requiring comprehensive clinical data demonstrating safety and effectiveness while creating new device classifications for future 510(k) submissions. FDA requires age-specific clinical validation when adult data are insufficient to predict pediatric safety and effectiveness, including enhanced safeguards under 21 Code of Federal Regulations (CFR) Part 50 Subpart D (Additional Safeguards for Children in Clinical Investigations), Institutional Review Board (IRB) review with pediatric expertise, and four-category risk assessment for pediatric studies [22]. The regulatory framework emphasizes the unique vulnerabilities of pediatric populations and requires additional ethical considerations beyond standard adult clinical trials. In contrast, European Union approaches have generally required less stringent clinical evidence compared to the FDA, with most DTx receiving Class I or IIa certification; however, ongoing updates under the EU Medical Device Regulation (MDR) since 2021 are gradually raising evidence standards and may bring EU requirements more closely in line with FDA pathways. EndeavorRx has Conformité Européenne (CE) marking in Europe; however, labeling and age indications may differ from those in the United States [23]. The international regulatory landscape continues to evolve as different jurisdictions develop frameworks specific to digital therapeutics.

Implementation challenges in pediatric digital therapeutics

Technology Access and Digital Divide

Digital poverty represents a significant barrier to equitable pediatric DTx access, with rural and socioeconomically disadvantaged families facing challenges including unreliable internet connectivity, lack of suitable devices, and data costs. Engagement with digital interventions varies significantly by developmental stage and technology access, while geographic disparities affect access to technical support and digital literacy resources. Device compatibility requirements across smartphones, tablets, and computers increase development complexity and implementation barriers [24]. The digital divide is particularly pronounced in pediatric populations, where family resources and technological infrastructure directly impact treatment access. Schools and community centers may serve as important access points for digital therapeutics, but this requires coordination between healthcare providers and educational institutions to ensure continuity of care. While evidence on formal school-based partnerships in pediatric DTx remains limited, emerging models from school-based mental health programs demonstrate the potential for integrating digital platforms into educational settings, though further research is needed to establish best practices [25].

Healthcare Integration and Workflow Challenges

Provider adoption faces persistent barriers, including limited digital literacy among healthcare professionals, unclear integration pathways within existing clinical workflows, and a lack of standardized e-prescribing protocols for DTx. Healthcare providers have expressed enthusiasm for digital interventions, though some remain cautious about their positioning relative to face-to-face treatment. Workflow disruption includes time burden for setup, monitoring, and support, limited integration with electronic health records, and unclear clinical decision support protocols. Training programs and the presence of digital champions within healthcare organizations are often described as important facilitators of successful digital health implementation [26]. The successful integration of DTx requires fundamental changes to clinical practice patterns and may necessitate new reimbursement models that account for provider time spent on digital health management. Many healthcare systems lack the technical infrastructure to support seamless DTx integration, requiring significant capital investment and staff training.

Reimbursement and Coverage Barriers

Fragmented payment landscapes lack centralized coverage models, creating inconsistent access across state Medicaid programs and commercial payers. Evidence requirements vary significantly across payers, with limited consensus on sufficient evidence thresholds for coverage decisions. Traditional health economic evaluation approaches may not capture the iterative nature and continuous improvement capabilities of digital interventions, necessitating novel outcomes measurement approaches and value-based contracting models [24]. Several U.S. state Medicaid programs have initiated pilot projects to evaluate coverage of prescription digital therapeutics, including ADHD-focused platforms, to assess feasibility and cost-effectiveness. In Europe, national health systems in countries such as Germany and the United Kingdom have introduced digital health formularies (e.g., Digital Health Applications (DiGA) in Germany) that provide reimbursement pathways for digital therapeutics, although pediatric-specific inclusion remains limited [27]. The lack of standardized billing codes for DTx creates an administrative burden for both providers and payers. Value-based care models may be particularly well-suited for DTx implementation, as these interventions can provide continuous monitoring and outcome tracking that supports risk-sharing arrangements between payers and providers.

Pediatric-Specific Implementation Considerations

Age-specific design requirements include interface considerations for fine motor skills, attention span limitations, and cognitive development stages, with content that must accommodate varying literacy levels and cultural considerations across diverse pediatric populations. Enhanced safety surveillance requirements include real-time monitoring capabilities, clear escalation protocols, and professional oversight requirements varying by age and condition. Children's Online Privacy Protection Act (COPPA) requirements for children under 13 mandate verifiable parental consent while balancing child autonomy with oversight needs [28,29]. The developmental nature of children requires continuous reassessment of appropriateness as they grow and mature. Family dynamics and caregiver involvement add complexity to implementation, as treatment success often depends on parent or guardian engagement and technological competency. Cultural sensitivity is particularly important in pediatric populations, as family values and communication patterns can strongly influence treatment acceptance and adherence. However, evidence on the impact of cultural tailoring in pediatric digital therapeutics remains limited, and further studies are needed to establish outcomes across diverse populations.

**Figure 1:**
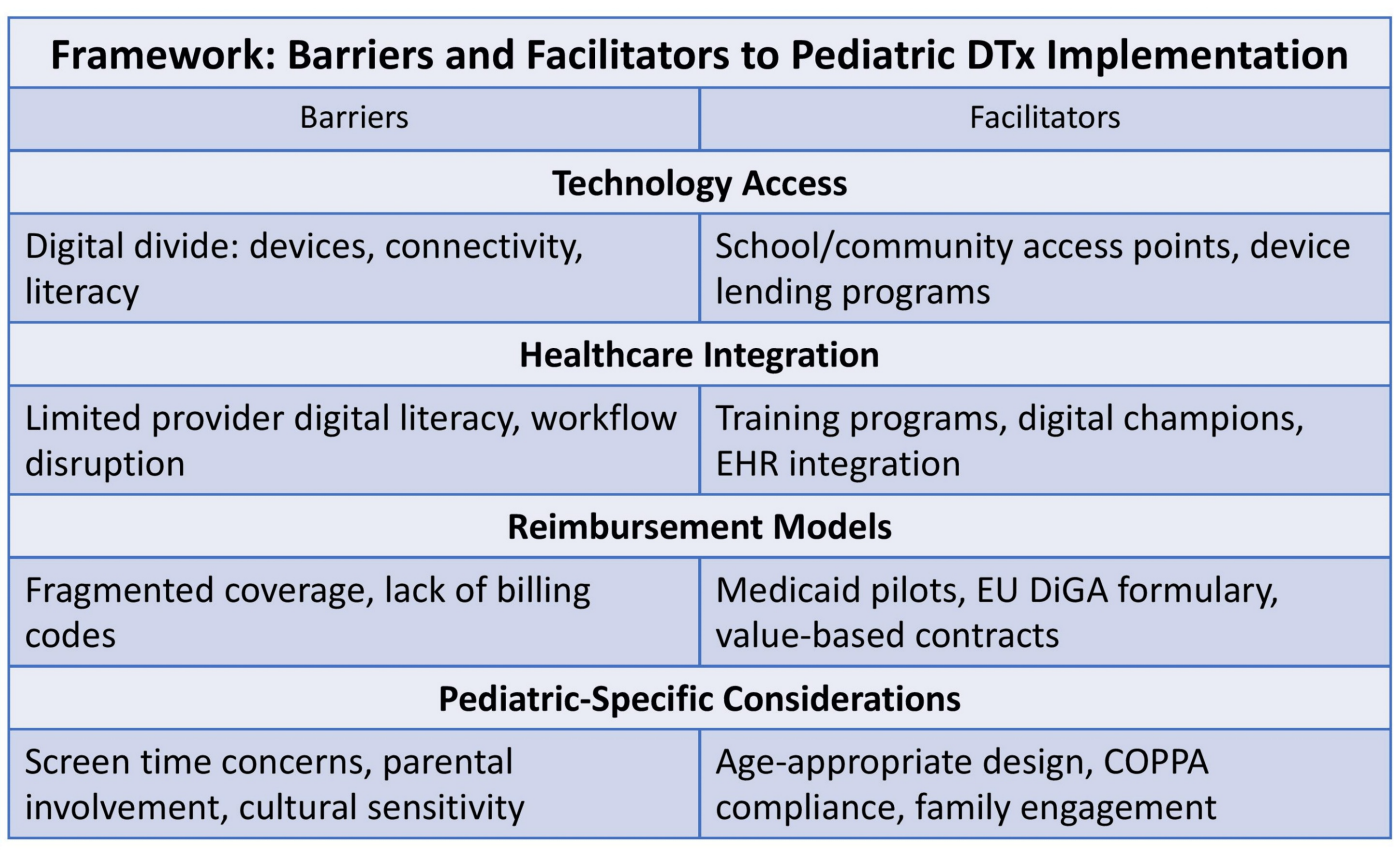
Framework of Barriers and Facilitators to Pediatric Digital Therapeutics Implementation This framework summarizes barriers and facilitators across four domains: technology access, healthcare integration, reimbursement models, and pediatric-specific considerations. Barriers include the digital divide (limited devices, internet connectivity, and digital literacy), limited provider digital literacy, fragmented reimbursement pathways, and concerns related to screen time, parental involvement, and cultural appropriateness. Facilitators include school- and community-based access, provider training and integration with electronic health records (EHRs), reimbursement initiatives such as U.S. Medicaid pilot programs and the European Union Digital Health Applications (DiGA) formulary, and age-appropriate design compliant with the Children’s Online Privacy Protection Act (COPPA) that incorporates family engagement. The image was created by author of this article, Venkata Sushma Chamarthi.

Figure 1 summarizes the barriers and facilitators to pediatric digital therapeutic implementation across four domains: technology access, healthcare integration, reimbursement models, and pediatric-specific considerations.

Future directions and emerging technologies

AI-Powered Personalized Interventions

AI integration enables real-time therapeutic adaptation based on individual patient responses and developmental stages. GuessWhat therapeutic game demonstrates pediatric facial emotion classification with >10% improvement over existing classifiers, while digital biomarker integration supports cognitive health assessment and treatment optimization. Machine learning algorithms facilitate predictive modeling for treatment outcomes, enabling personalized intervention selection and dosing optimization. Multi-modal AI combining visual, auditory, and behavioral inputs promises enhanced therapeutic precision and effectiveness [8,23]. However, personalization in pediatric digital therapeutics also faces challenges, including potential algorithmic bias due to under-representation of diverse ethnic and socioeconomic pediatric populations, which may limit generalizability and equity of outcomes [30]. Pediatric-specific data on AI bias remain limited, underscoring the need for inclusive datasets in this field. The integration of natural language processing allows for more sophisticated patient-AI interactions and can provide real-time sentiment analysis to adjust therapeutic approaches. Emerging modalities, such as vocal biomarkers, offer non-invasive, scalable tools for monitoring neurological, respiratory, and emotional health in pediatric populations, with potential integration into DTx platforms for early detection and real-time therapeutic adjustment [31]. These AI-powered systems can continuously learn from patient interactions, creating increasingly personalized treatment protocols that adapt to individual learning styles and preferences.

Virtual and Augmented Reality Applications

Virtual reality (VR) applications demonstrate significant potential across pediatric conditions, including mental health, neurodevelopment, and procedural support. DTx utilizing VR technology are being evaluated in pediatric procedural-anxiety programs at tertiary centers, with pilot randomized controlled trials showing significant anxiety reduction and improved procedural tolerance [32]. In autism spectrum disorder, virtual reality-based social cognition training programs have demonstrated improvements in emotion recognition, conversational skills, and social engagement metrics, although most published evidence to date comes from studies in young adult populations rather than children, highlighting the need for further pediatric-focused trials [33]. Additional applications include augmented reality (AR)-guided neurosurgical planning, immersive environments for social anxiety interventions, and educational VR platforms that create 3D anatomical visualizations to support patient education and treatment adherence. These immersive technologies offer engaging, interactive environments that may enhance therapeutic engagement, though evidence on the transfer of skills from VR to real-world contexts in pediatric populations remains limited and warrants further research. The controlled nature of virtual environments allows for systematic exposure therapy and skill practice without real-world consequences, making them particularly valuable for anxiety-based conditions and social skills training. Advanced haptic feedback systems are being integrated to provide tactile experiences that can enhance learning and retention of therapeutic concepts.

Wearable Technology Integration

Advanced biosensor development includes Wi-Fi-enabled surface electromyographic devices optimized for children with movement disorders, cardiac monitoring integration with consumer devices, and smart clothing with embedded sensors for continuous health monitoring [23]. Wearable integration with gaming platforms enhances therapeutic engagement while providing passive monitoring of activity, sleep, and physiological parameters for treatment optimization and safety monitoring [10]. The miniaturization of sensor technology has enabled the development of child-friendly wearables that can monitor multiple physiological parameters without interfering with daily activities. However, their use also raises important considerations regarding pediatric data privacy, including potential breaches and regulatory complexities under frameworks such as the European Union’s General Data Protection Regulation (GDPR) and the U.S. COPPA. Integration with DTx platforms allows for real-time biofeedback and adaptive therapeutic responses based on physiological state. Advanced algorithms can detect patterns in wearable data that may predict treatment response or identify early signs of clinical deterioration, enabling proactive intervention adjustments.

Pipeline Developments and Investment Landscape

The pediatric DTx sector continues to expand with major companies, including Akili Interactive and other developers, expanding beyond current approvals with pipeline programs targeting depression, autoimmune disorders, and pain management [4-5]. Clinical trials in pediatric DTx focus on developmental appropriateness and long-term safety assessment through specialized research networks [3]. The investment landscape shows increasing venture capital interest in pediatric-focused DTx companies, driven by the substantial unmet need and potential for global scalability. Pharmaceutical companies are increasingly partnering with DTx developers to create combination therapies that integrate digital and pharmacological interventions. The pipeline includes novel therapeutic areas such as pediatric sleep disorders, eating disorders, and rare genetic conditions, demonstrating the expanding scope of digital therapeutic applications in pediatric medicine.

Limitations

This review has several limitations. First, it is not a systematic or scoping review; as such, PRISMA-style reporting could not be applied. Although a structured search strategy across major databases was employed, selection bias remains possible, and some relevant studies may have been missed. Because the review was designed as a narrative synthesis, effect sizes, sample sizes, and direct comparisons with conventional therapies were not systematically extracted; instead, the aim was to provide a broad and accessible overview, with references included for readers seeking more detailed data. Second, the current evidence base for pediatric digital therapeutics remains limited, with the most robust data confined to ADHD and amblyopia, while other conditions rely on small pilot studies or preliminary trials. Third, long-term safety, durability of treatment effects, and cost-effectiveness have not been comprehensively evaluated in pediatric populations. Finally, given the rapid pace of technological innovation and evolving regulatory frameworks, findings may become outdated quickly. These limitations highlight the need for future systematic reviews and ongoing real-world evidence generation to strengthen the evidence base for pediatric digital therapeutics.

## Conclusions

Pediatric digital therapeutics represent a transformative approach to addressing significant gaps in children's healthcare access and clinical outcomes. They demonstrate broad potential across multiple conditions, with early innovations suggesting a foundation for future regulatory pathways. Emerging approaches are being explored in areas such as neurodevelopmental, behavioral, and chronic health conditions, showing encouraging early signals of effectiveness. However, successful widespread implementation requires addressing unique pediatric considerations, including developmental appropriateness, family dynamics, digital equity, and specialized safety requirements. Key success factors include meaningful stakeholder engagement incorporating children, families, and healthcare providers; integrated care pathway positioning rather than standalone solutions; and systematic approaches to evidence generation addressing iterative digital intervention characteristics. The regulatory framework continues evolving, with the FDA providing clear approval pathways while maintaining rigorous safety standards appropriate for vulnerable pediatric populations.

Future directions emphasize AI-powered personalization, VR/AR therapeutic applications, advanced wearable integration, and family-centered ecosystem approaches. Addressing implementation barriers, including reimbursement models, provider training, and equitable access, will be critical to realizing the transformative potential of pediatric digital therapeutics in improving health outcomes for children and adolescents. Organizations implementing pediatric DTx should prioritize co-design methodologies, multi-stakeholder engagement strategies, and continuous real-world evidence collection to ensure both clinical effectiveness and equitable access across diverse pediatric populations. The convergence of advanced technologies with child-centered design principles promises therapeutic interventions that are clinically effective, engaging, accessible, and developmentally appropriate for young patients and their families.
